# Osmodehydrofreezing of Tomatoes: Optimization of Osmotic Dehydration and Shelf Life Modeling

**DOI:** 10.3390/foods13172689

**Published:** 2024-08-26

**Authors:** Efimia Dermesonlouoglou, Lefteris Pittas, Petros Taoukis, Maria Giannakourou

**Affiliations:** Laboratory of Food Chemistry and Technology, School of Chemical Engineering, National Technical University of Athens, 15780 Athens, Greece; efider@chemeng.ntua.gr (E.D.); lefteris2352000@gmail.com (L.P.); taoukis@chemeng.ntua.gr (P.T.)

**Keywords:** cherry tomato, osmodehydrοfreezing, process optimization, frozen storage, quality loss, shelf-life

## Abstract

The objective was to review, using an integrated approach, all parameters related to osmotic dehydration, freezing, and frozen storage when assessing the advantages of the osmodehydrofreezing-ODF process. Peeled cherry tomatoes were treated at (T) 25, 35, and 45 °C (t) up to 180 min in glycerol-based OD-solution (50, 60, 70% *w*/*w*). OD was studied and optimized by applying the Response Surface Methodology, combined with selected desirability criteria to define the optimum process parameters. Water loss-WL, solid gain-SG, water activity reduction-a_w_, texture and color changes were monitored during the process. Untreated and OD-treated at optimal OD conditions (C = 61.5%, T = 36 °C; t = 72 min) samples were frozen and stored at isothermal (T, −5, −8, −14, −23 °C) and non-isothermal temperature conditions (T_eff_, −7.3 °C). OD samples presented acceptable color, increased firmness, low drip loss and high vitamin C/lycopene retention during frozen storage. OD increased the shelf life of frozen cherry tomato (up to 3.5 times based on sensory quality loss). The kinetic models obtained for vitamin and lycopene degradation and sensory quality loss were validated at non-isothermal conditions.

## 1. Introduction

Osmotic dehydration (OD) entails submerging food products in hypertonic aqueous solutions that contain carbohydrates and/or salts to partially remove water without causing a phase change [1]. Mass transfer occurring during the OD process involves the water transfer from the product matrix to the osmotic solution, the solute transfer (taking place in opposite directions), and, to a lesser extent, the leaching of the product’s solutes [2]. The goal of osmotic processing is to reduce the water activity of the product without significantly altering its quality, unlike other dehydration methods (such as air-drying) [2,3]. Food material characteristics, molecular weight and concentration of the solutes in the osmotic solution, the temperature of the process, the length of immersion, and the agitation level affect the rate of mass transfer [4,5,6,7,8,9,10]. Furthermore, studies have been carried out to better understand mass transfer and model the mechanisms of the OD process [11,12]. Especially for plant-based food, such as fruits and vegetables, that are susceptible to significant tissue damage when high temperatures are applied, OD is found to lead to improved quality retention, particularly when compared to other, more intense dehydration techniques like air-drying [13,14]. However, osmotically dehydrated fruits and vegetables do not reach a water activity level to render them microbiologically stable and a complementary preservation method such as freezing or air-drying is required to obtain an extended shelf life. Besides lowering food water activity and thus improving its stability, OD could be also used as a tool in matrix engineering since it typically results in the selective uptake of solutes, such as bioactive components, components targeting texture, flavor, or nutritional value enhancement [4,15,16].

Osmodehydrofreezing (ODF) is the application of partial dehydration in a hypertonic solution prior to a subsequent traditional freezing procedure [17]. The application of osmotic pre-treatment before conventional freezing lowers the freezing point and significantly reduces the water amount to be frozen thereafter. When a solute is impregnated and the moisture content decreases, the glass transition temperature of the modified tissue rises, greatly increasing its stability in the subsequent frozen storage [11,12]. Osmodehydrofreezing has several positive effects on the freezing process itself, such as better preservation of structure and other quality attributes, lower packaging, distribution, and storage costs, lower energy to freeze the remaining water and a decrease in the refrigeration load [18]. Despite the above-mentioned advantages, ODF’s wider exploitation and industrial application are practically limited [2]. This is primarily because it is challenging to model and control mass transport phenomena, additionally due to issues related to osmotic solution management [19,20]. In this context, there is a need to implement an integrated kinetic approach aiming at optimizing the ODF, using criteria such as lowering the product water activity as well as the quality loss reaction rates. Response surface methodology (RSM) has been frequently proposed as a means of characterizing and pinpointing the primary elements impacting processes. Moreover, the desirability approach is used, as a complementary RSM tool, to optimize multiple responses, when there is a need to apply multiple optimization criteria [21] and this strategy has been recently implemented for optimizing the osmotic dehydration process [6,7,16,22,23,24].

Cherry tomato is abundant in nutrients and bioactive compounds, and recently, an economically important commodity in multiple recipes [25]. Its small size, tomato-like flavor, and variety of colors make it a favorite option. Cherry tomatoes in different colors and tastes are bought and eaten raw; however, they can also be found in processed foods [26]. Its thin skin and juicy characteristics make it perishable during storage and transportation, [27,28]. Therefore, it is necessary to find suitable processing and/or packaging methods to improve the storage life of cherry tomatoes. Freezing, as a single preservation method, gives poor results, especially when frozen tomatoes are used for direct consumption [29].

In this study, the quality of osmo-dehydrofrozen (produced at optimized OD conditions using RSM, coupled with selected desirability functions) cherry tomatoes was kinetically studied during frozen storage. More specifically, mass exchange, color and texture changes were monitored during the OD process. After processing tomato pieces at the optimal OD conditions, samples were frozen and packed. Vitamin C and lycopene, color, texture, drip loss and overall acceptability of OD pre-treated as well as conventionally (untreated) frozen samples were measured during storage at isothermal and non-isothermal temperatures at a wide range of sub-zero temperatures and product shelf life was calculated. Applying this integrated process (ODF), involving three steps (OD-freezing-frozen storage), one can better assess its advantages, compared to the conventional freezing preservation, in terms of the stability and the improved shelf life of the ODF tomato samples, obtained.

## 2. Materials and Methods

### 2.1. Sample Preparation

Cherry tomatoes (*Lycopersicon esculentum*) (diameter: 30 ± 10 mm) were purchased at the local market and kept in temperature-controlled cabinets for a maximum of 1 day (approximately 4.0 °C). Before osmotic treatment, tomatoes were washed with tap water, and then, blanched in water at 80 °C for 30 s, for both peel removal and enzyme inactivation. Water activity (a_w_) was measured at 0.9505 ± 0.0076, total soluble solids (TSS) were 8.8 ± 0.6 °Brix and pH was 4.108 ± 0.012 (mean ± standard deviation; five samples). According to Gould (2013), all measured values fall within the acceptable limits for ripeness [30]. Striking is the high content of total soluble solids (approximately 7.0 °Brix) which is commonly perceived as a good predictor for both ripeness and high sensory quality [24,25].

### 2.2. Osmotic Dehydration

Osmotic dehydration (OD) was carried out in solutions by dissolving glycerol (Glycerine EP 212, OLEOGEN, Genova, Italy), (C) 50, 60, and 70% (*w*/*w*), 3.5% NaCl and 1.5% CaCl_2_, for (t) up to 180 min at (T) 25, 35, and 45 °C [8]. to yield a solid to liquid ratio of 1:5 (*w*/*w*). Glycerol was used as the main lowering a_w_ agent sugar alcohol with low sweetness (Regulation EC No. 1333/2008) [31]. Additionally, it acts as a cryoprotectant and it improves the texture. Sodium chloride was included in the osmotic solution to balance the slight sweetness obtained during osmotic pretreatment as well as to increase the driving force of the process [32]. Calcium chloride was used to retain or even to improve the texture properties of the osmodehydrated product. Calcium may interact with cellular pectins and other wall components modifying the structural and mechanical properties of plant tissue [33]. Tomatoes were pre-weighed, put in cylindrical glass containers and placed in water baths of controlled temperature, under constant agitation (240 rpm). At times (t) 15, 30, 60, 90, 120, 150, and 180 min samples (triplicates) were removed from jars and weighed after carefully blotting the excess coating solution.

#### 2.2.1. Water, Total Solids and Water Activity

Water content and total solids were monitored during the process, measured by drying at 105 °C for 24 h according to the AOAC method (1990). Water activity (a_w_) was monitored using Aqua LAB 4TEV (Decagon Devices, Inc., Pullman, WA, USA).

#### 2.2.2. Mass Transfer

Water loss (*WL*) and solid gain (*SG*), which describe the mass transfer phenomena during the OD process, were calculated (Equations (1) and (2)) [34]:

(1) $$WL=\frac{\left(M_{0}-m_{0}\right)-\left(M-m\right)}{m_{0}}$$

(2) $$SG=\frac{\left(m-m_{0}\right)}{m_{0}}$$

where *M*_0_ and *m*_0_ are the initial total mass and dry mass of the tomato sample at OD time zero, respectively, and *M* and *m* are the total mass and dry mass of the tomato sample at OD time *t*, respectively.

#### 2.2.3. Experimental Design and RSM Implementation

The optimization of the OD process was performed by applying a symmetrical 15-run three-level Box–Behnken design (BBD). Temperature (x1), 25, 35, 45 °C; time of osmotic treatment (x2), 30, 60, 90 min and glycerol concentration (x3), 50, 60, 70% were the factors under optimization or independent variables, while mass exchange (*WL*, *SG*), water activity (a_w_), texture (firmness), and color (Δ*E*) changes were the response of the model or dependent variable, which were adequately modeled using a second-order polynomial model describing the effect of the most important OD processing factors. Since the examined factors have different natural units, their actual values should be transformed into dimensionless normalized coded units (−1, 0, +1) to directly compare and evaluate their effect and the effect of their interactions on the dependent variables. The actual and coded values of the investigated extraction factors are presented in Table 1.

#### 2.2.4. Factor Interactions during Osmotic Dehydration

A second-order polynomial was used to describe the interactions between response variables Y (water loss WL and solid gain SG, water activity a_w_) and the factor variables X_i_ (concentration of glycerol co C) 50, 60, 70%, process temperature (T) 25, 35, 45 °C, and process time (t) 5–180 min (Equation (3)) [35].

(3) $$y=a_{0}+\sum a_{i}x_{i}+\sum a_{ii}{x_{i}}^{2}+\sum a_{ij}x_{i}x_{j}\;$$

where *a*_0_ is the constant, *a_i_* is the linear, *a_ii_* is the quadratic, and *a_ij_* is the interaction effects of the factors. The quadratic variables indicate the surface curvature, the linear variables the coordinates of the greatest value anticipated, and the bi-factorial cross-variables the directions of the axes of the geometric figure formed by sectioning the surface area [35]. Positive values of the coefficient indicate an effect that favours the response, whereas negative values show an opposite effect of the factor on the response. After assessing the polynomial equations’ coefficients and calculating the model, analysis of variance (ANOVA) was applied to investigate how well the model describes the data. The corresponding *p* value served as a statistical significance indicator for this purpose.

### 2.3. Freezing Process

Cherry tomatoes, osmodehydrated and non-osmodehydrated (untreated), were quick frozen at −40 °C (forced air convection characteristics, h = 11 W/m^2^K) (Sanyo MIR 553, Sanyo Electric Co., Gunma, Japan), packed in pouches used for commercial frozen vegetable products [laminate film characteristics: 20 lm bio-oriented polypropylene (BOPP)-48 lm polyethylene (PE), water-vapor transmission rate (WVTR) < 6 g/m^2^ d], and kept at −40 °C.

### 2.4. Storage at Sub-Freezing Temperatures

Osmodehydrated and non-osmodehydrated (untreated) cherry tomatoes were stored in temperature-controlled cabinets (Sanyo MIR 153, 253, Sanyo Electric Co., Gunma, Japan) at isothermal temperatures (T) −5, −8, −14, −23 °C (±0.5 °C), monitored by temperature dataloggers (COX TRACER^®^, Belmont, NC, USA). The experimental set-up was according to ASLT (Accelerated Shelf-Life Testing) principles [36]. Quality and sensory parameters (color, texture, drip loss, vitamin C, lycopene) of osmodehydrofrozen (OD) and non-osmodehydrated (untreated) frozen cherry tomato samples were determined at predetermined time intervals during storage based on a rough estimate of an expected temperature-dependence of quality loss [4].

The applicability of the developed (at isothermal conditions) kinetic models was tested by an independent experiment (at non-isothermal conditions). A temperature-time scenario was applied, by repeating three successive cycles of −12 °C–24 h, −5 °C–24 h and −8 °C–24 h (Sanyo MIR 153, Sanyo Electric Co., Gunma, Japan) equal to an effective temperature (*T_eff_*) of −7.3 °C [37].

#### 2.4.1. Color

Tomato color, expressed in CIELab scale, was measured by a chromameter (CR-200, which uses the D65 illuminant and a 10-degree observer for its measurements, Minolta Co., Tokyo, Japan). The total color change during the OD process as well as during the frozen storage, was mathematically described by Equation (4) [38].

(4) $$∆E=\sqrt{{\left(L-L_{0}\right)}^{2}+{\left(a-a_{0}\right)}^{2}+{\left(b-b_{0}\right)}^{2}}\;$$

where *L* (lightness), *a* (from +: red to −: green), *b* (from +: yellow to −: blue), and $L_{0},\;a_{0},\;b_{0}$ values at time t, and at time zero (0), either of the OD process or of the frozen storage, respectively.

#### 2.4.2. Texture

Texture measurements of cherry tomato samples (OD and untreated), equilibrated to room temperature for 30 min, were measured by a texture analyzer (TA-XT2i of Stable Micro Systems, England). The test took place on a non-lubricated flat platform with a knife probe, used for cutting samples at a test speed of 1 mm/s and a depth of 1/3 of the initial (approximately 5 mm). The maximum peak force (*F_max_*, g) was reported as hardness/firmness. Eight to ten samples (3 replicates) were used.

#### 2.4.3. Drip Loss

Osmodehydrated and non-osmodehydrated (untreated) cherry tomatoes were put on a sieve to allow their exuded water to drip off and then left to defrost at room temperature for 60 min. Samples were weighed at time intervals of 10 min. The difference between the weight at any time t and the initial weight referred to as the initial value was defined as the drip loss of the sample [39] (Equation (5)).

(5) $$Drip\;loss\;\left(\%\right)=\frac{W_{before\;thawing}-W_{after\;thawing}}{W_{before\;thawing}}*100$$

where *Wi*, (g) the weight of tomato before freezing and $W_{after\;thawing}$ the weight of the tomato after freezing.

#### 2.4.4. Vitamin C

Vitamin C (mg L-ascorbic acid/100 g fresh/OD material) was determined by a high-performance liquid chromatography method (HPLC) [37]. Two replicates were used. Five g of osmodehydrofrozen or untreated sample homogenates were mechanically stirred in 15 mL of a 4.5% (*w*/*v*) solution of metaphosphoric acid (Merck, Darmstadt, Germany) for 15 min (two replicates for each measurement). The mixture was filtered under vacuum and diluted using HPLC grade water (Merck, Darmstadt, Germany); the total final volume was recorded and an aliquot was filtered through a 0.45 m filter (Chromafil PVDF-45/25, Macherey-Nagel, Germany) prior to injection into the chromatographic column. The details of the HPLC instrumentation were HP Series 1100 (quarternary pump, vacuum degasser, a Rheo dyne 20 μL injection loop and a Diode-Array Detector, controlled by HPChemStation 1100 series software); Hypersil ODS column (250 × 4.6 mm) of particle size 5 μm; mobile phase: HPLC grade water with metaphosphoric acid to pH 2.2; detection at 245 nm; calibrated by external standard method.

#### 2.4.5. Lycopene

Lycopene (mg total lycopene/100 g fresh/OD material) was determined using a high-performance liquid chromatography method [40]. Two and a half g of osmodehydrofrozen or untreated sample homogenates were added to a mixture consisting of 50 mL of hexane, 25 mL of ethanol, 25 mL of acetone and 0.05% (*w*/*v*) butylated hydroxytoluene, then stoppered and mechanically agitated for 5 min (two replicates for each measurement). Cold, doubly distilled water (10 mL) was added and the suspension was agitated for an additional 5 min. The solution was allowed to stand for 15 min for separation of polar and non-polar layers. The upper hexane layer, containing the lycopene, was filtered through a 0.22-lm filter (Chromafil PET-20/25; Macherey-Nagel, Duren, Germany) and then injected into the chromatographic column. The instrumentation was the same as in Section 2.4.4. The mobile phase was methanol:tetrahydrofuran:water at a ratio of 67:27:6. The detection was performed at 472 nm. The method was calibrated by an external standard method.

#### 2.4.6. Sensory Parameters

Sensory evaluation was carried out by a panel of eight to ten trained assessors [70% female and 30% male, 60% young (18–35 years old) and 40% middle-aged (36–55 years old)] of the sensory laboratory of NTUA, according to ISO standards [41,42] (ISO 6658:2017, ISO 20613:2019), on the following attributes: appearance for red color; firmness and juiciness for texture, sweetness, sourness for flavor and aftertaste. The intensity was evaluated using a 9-point scale (1 = low intensity and 9 = high intensity). The panel also performed an overall impression test using a 9-point hedonic scale (1 = extremely dislike to 9 = extremely like).

#### 2.4.7. Data Analysis

The evolution of the selected quality and sensory parameters of osmodehydrofrozen (OD) and non-osmodehydrofrozen (untreated) cherry tomato samples were plotted and mathematically modeled, at all studied temperatures; The quality loss rates *k* were calculated. The vitamin C (ascorbic acid) and lycopene loss were described by an apparent first-order reaction (Equation (6)) [43], whereas the sensory quality loss was described by an apparent zero-order reaction (Equation (7)) [6].

(6) $$C_{\frac{asc}{lyc}}=C_{\frac{asc}{lyc}0}\;\cdot \;exp\left(-k_{\frac{asc}{lyc}}\cdot t\right)$$

(7) $$\;S_{overall}{=S}_{overall\;0}-k_{sens}\cdot t$$

where *C_asc/lyc_* and *Casc_/lyc_*_0_ are the concentrations of vitamin C or lycopene at time t and zero, respectively, *S_overall_* and *S_overall,_*_0_ are the sensory scores at time t and zero, respectively, and *k_asc/lyc_, k_sens,overall_* is the apparent reaction rate of vitamin C loss, lycopene loss or overall sensory quality loss.

The temperature dependence of the deterioration rates *k_asc/lyc_/k_sens,overall_* was modelled by the Arrhenius equation (Equation (8)) [36]:

(8) $$k_{\frac{asc}{lyc},overall}{=k}_{ref}\;\cdot \;exp[-\frac{E_{a}}{R}(\frac{1}{T_{ref}}-\frac{1}{T})]$$

The shelf life, determined (based on the vitamin C or lycopene loss), was calculated using Equations (6) and (8) (Equation (9)). The shelf life (*SL*), determined by the overall sensory quality, was calculated using Equations (7) and (8) (Equation (10)):

(9) $$SL_{asc/lyc}=\frac{lnC_{\frac{asc,final}{lyc,\;final}}-lnC_{\frac{asc}{lyc},0}}{k_{{asc/lyc}_{Tref}}\exp\left(-\frac{E_{a_{asc/lyc}}}{R}\left(\frac{1}{T}-\frac{1}{T_{ref}}\right)\right)}$$

(10) $$SL_{s}=\frac{S_{0}-S_{L}}{k_{s_{Tref}}\exp\left(-\frac{E_{a_{s}}}{R}\left(\frac{1}{T}-\frac{1}{T_{ref}}\right)\right)}$$

where *E_a asc/lyc_* is the activation energy of the parameter *k*, *k_Tref_* is the deterioration rate at the reference temperature *T_ref_* (−18 °C, in this study), *R* is the universal gas constant, $C_{\frac{asc,final}{lyc,final}}$ is the acceptance limit of vitamin C/lycopene content (50% loss) [37,44], *S_L_* is the acceptance limit of the sensory parameter *S* (overall impression score = 5) [8]. Using these equations, the shelf life can be predicted at different storage temperatures (in the same temperature range). In the case of variable temperature profiles, common for temperature abuse during real conditions, where T becomes a function of time (T(t)), Equations (7) and (8) are transformed to integrals, and accordingly solved to obtain *SL* decrease at each iso-thermal time step.

Variable temperature profiles were expressed by the effective temperature concept [37]. Effective temperature *T_eff_*, is defined as the constant temperature leading to the same quality parameter value as the variable temperature distribution (that can be discretized into small isothermal time steps, *t_i_*) over the same time period, is based on the Arrhenius model and integrates into a single value the effect of the variable temperature profile as given in Equation (11),

(11) $$k_{ref}\sum_{i}\left(\exp\left[-\frac{E_{A}}{R}\left(\frac{1}{T_{i}}-\frac{1}{T_{ref}}\right)\right]t_{i}\right)=k_{eff}t_{tot}=k_{ref}\cdot \exp\left[-\frac{E_{A}}{R}\left(\frac{1}{T_{eff}}-\frac{1}{T_{ref}}\right)\right]t_{tot}$$

from which the value of *T_eff_* can be calculated.

The rates of quality deterioration calculated by the developed kinetic models were compared to the respective experimental values by calculating the relative error (*RE*) values (Equation (12),

(12) $$\%RE=\frac{k_{exp}-k_{fitted}}{k_{exp}}\;\cdot \;100$$

where 20% is the limit of acceptance [43].

### 2.5. Statistical Analyses

Analysis of variance (ANOVA) was conducted by STATISTICA 12.0 software (Stat. Soft. Inc. Tulsa, OK, USA), while significant differences in mean values of WL, SG, and a_w_ values were estimated by Tukey’s HSD test (*p* < 0.05). Regarding RSM implementation and desirability approach, the data processing and the statistical analysis were performed using Minitab 17 software (trial version, Minitab LCC, State College, PA, USA).

## 3. Results and Discussion

### 3.1. OD Process

#### 3.1.1. Mass Exchange during OD Process

**Figure 1:**
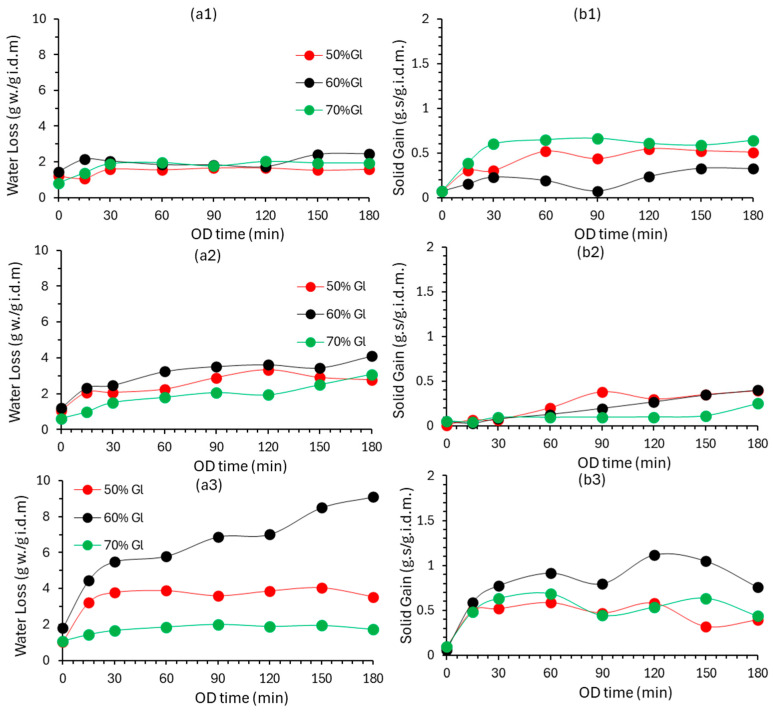
Evolution of (**a**) water loss (WL), and (**b**) solid gain (SG) of OD cherry tomatoes at (**1**) 25, (**2**) 35, and (**3**) 45 °C using 50, 60, 70% *w*/*w* glycerol-based solution. (Experimental data points: average ± standard error).

The results for the OD of cherry tomato samples (50–70% *w*/*w* glycerol concentration and temperatures 25–45 °C) in terms of water loss (*WL*) and solid gain (*SG*) are presented in Figure 1a,b. According to the particular Figures, the dehydration (water loss) as well as solids uptake was accelerated with temperature increase. At OD time t = 0, i.e., the immersion time, there is an almost instantaneous water loss, as the mass transfer starts as soon as the sample meets the osmotic solution (maximum concentration gradient, which is the driving force for OD). The water loss rate increases during the first 30–60 min of the process; then it decreases and tends to stabilize, as reported by Ma et al. (2021) [45]. This effect had been reported for kiwi fruit which was subjected to osmotic dehydration with glycerol and sucrose. The high mass transfer in the early phase of osmotic treatment was due to the large osmotic pressure difference between the apple cube and the surrounding osmotic solution [46]. Glycerol concentration appears to have a significant effect on water loss, which assumes its maximum value for 60% glycerol concentration. Higher glycerol concentration (70%) probably prevents the OD solution components (NaCl, CaCl_2_) from dissolving completely, as reported by [13,47]. Figure 1b shows the uptake of solids (SG), owing to the transport of glycerol and salts inside the cells during OD, but also to the retention of components on the surface of the plant tissue during immersion. The solids uptake rate is observed to be increased at the beginning of the process (30–60 min). It seems that the temperature increase from 25 °C to 35 °C does not significantly affect the uptake of solids, while the increase from 35 °C to 45 °C significantly increases the solids uptake (almost double) [4].

#### 3.1.2. Water Activity during OD Process

**Figure 2:**
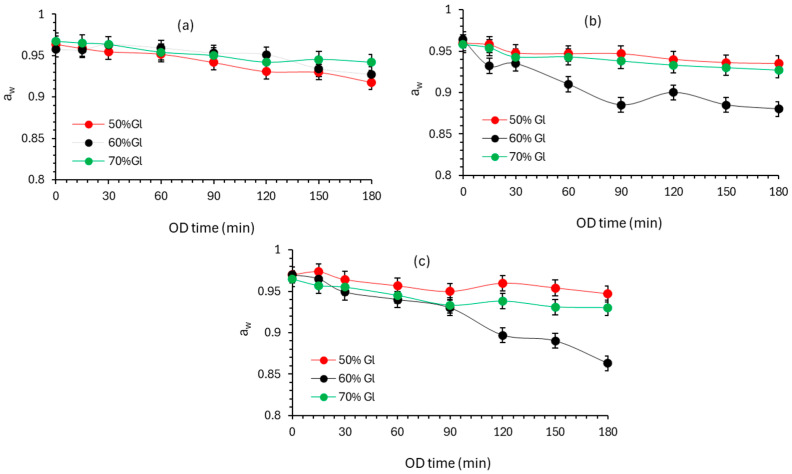
Evolution of water activity of cherry tomatoes during OD at (**a**) 25 °C, (**b**) 35 °C, and (**c**) 45 °C using 50%, 60%, 70% *w*/*w* glycerol-based solution. Markers indicate experimental data points (average ± standard deviation).

Water activity values obtained for OD tomato samples are plotted vs. OD time for all glycerol concentrations and temperatures studied (Figure 2a–c). As expected, water activity immediately decreases during the first minutes of the process and throughout its duration. According to Figure 2a–c, the temperature significantly accelerates water activity decrease (*p* < 0.05), in agreement with published work [37]. A significant a_w_ reduction seems to be achieved at 45 °C and 60% glycerol solution (t = 180 min => a_w_ = 0.85). When glycerol concentration increases, the rate of a_w_ decrease seems to increase, with 60% glycerol solution leading to the highest a_w_ decrease. In published studies [13,48], it was reported that the high carbohydrate concentration (in the osmotic solution) may present obstacles in the dissolution of the remaining components in the solution; for this reason, a lower water activity reduction rate is observed for the solution with 70% glycerol concentration (compared to 50 and 60%).

#### 3.1.3. Color and Texture Changes during OD

**Figure 3:**
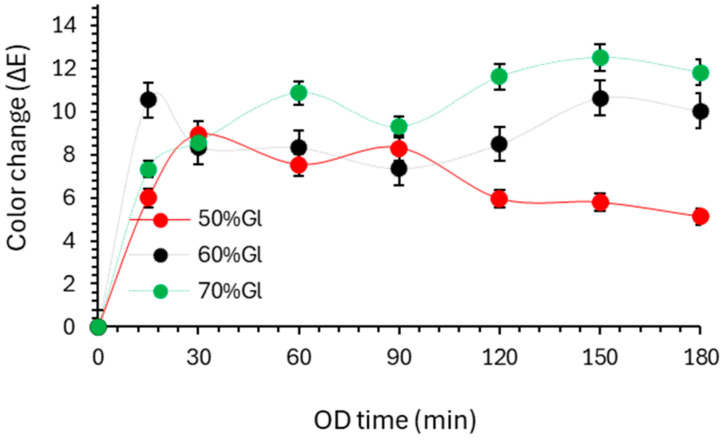
Evolution of color (Δ*Ε* index) of cherry tomato samples during OD (with temperature = 35 °C), using 50%, 60%, 70% *w*/*w* glycerol-based solution. Markers indicate experimental data points and error bars indicate the standard deviation from replicates.

In Figure 3, the total color change (Δ*E*) and texture (firmness, *F_max_*) of the osmodehydrated (OD) cherry tomato samples during OD at 35 °C is representatively depicted. It was observed that OD temperature (from 25 to 45 °C) does not affect significantly color change. For the OD temperatures studied, the color change rates were increased in the early stages (approximately during the 15–60 min) where the mass transfer was higher. Changes in tissue structure (semipermeable membrane in plant cells) caused by OD also affect the color change. During the osmotic dehydration process, carotenoids that are responsible for the color may diffuse into the solution and cause a partial loss of color [49]. The highest Δ*Ε* values were calculated for glycerol concentrations of 60 and 70%. The glycerol concentration seemed to have a significant effect on the color change, after 120 min of OD, for the 50–70% glycerol concentration. A *L* value decrease (lightness), along with a *b* value increase (yellowness) and *a* value increase (redness), was observed as OD time increased. Overall color Δ*Ε* values ranged from 6 to 12, expressing a significant perceivable color change of tomato samples. According to the interpretation by Choi et al. (2002), the Δ*E* value > 2 indicates the visible difference [50]. The perceived color change of tomatoes observed during OD could not be only connected to their pigment color but also to the spatial distribution of the pigments within the plant cells, which can change as the cells become more permeable and pigments are released from the chromoplasts into the cytoplasm. Even though there is some loss of dry matter towards the osmotic solution during OD (observed by slight red pigmentation of the solution post-treatment), the removal of water significantly counteracts it, leading to a concentration of the pigments in tomato samples [8].

**Figure 4:**
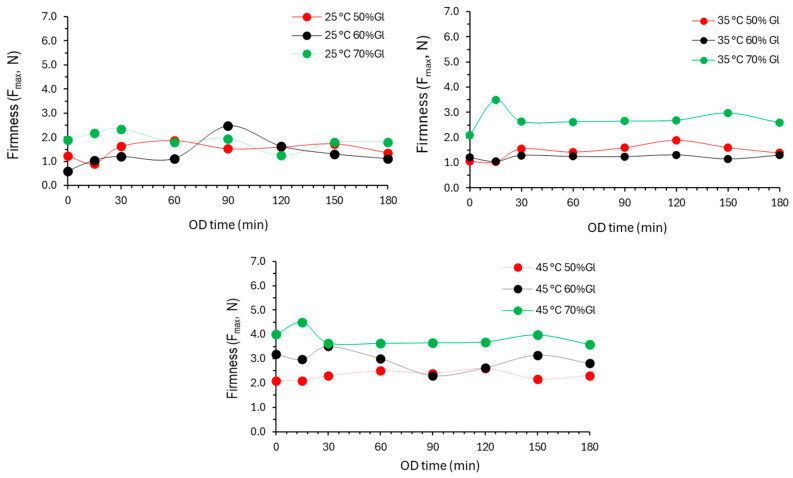
Evolution of texture (hardness, *F_max_*, N) of cherry tomato samples at the three different temperatures studied, namely 25, 35 and 45 °C, using, in each case, the three alternative osmotic solutions, containing 50%, 60%, and 70% *w*/*w* glycerol. Markers indicate experimental data points.

Regarding tomato texture during OD, the firmness was significantly affected by the OD temperature (Figure 4). The highest firmness values were calculated for 70% glycerol concentration. The 50 and 60% glycerol concentrations did not affect significantly the texture of the osmodehydrated tomato. Ninety-one hundred twenty (90–120)% higher firmness values were reported for osmotically dehydrated tomatoes compared to the non-osmodehydrated ones by Dermesonlouoglou et al. [4]. Nowacka et al. (2014) observed that the OD kiwifruits presented a decrease in firmness during the 60 min of OD, followed by an increase after 120 min [51].

### 3.2. OD Process Optimization

According to the results presented in Table 2, it could be concluded that the fitted models adequately represented the data and could be used to describe the design space. The asterisk sign in the table accounts for *p* value < 0.05 and shows which coefficients (contribution of each factor: linear, quadratic, interaction) are statistically significant, at a confidence level of 95%.

Water loss (*WL*) and solid gain (*SG*) values are influenced mostly by temperature (a1), as shown by the higher values of the corresponding factors (Table 2), and much less by osmosis time (a2) and glycerol concentration (a3); nonetheless, none of the process conditions of time (a2) and glycerol concentration (a3) has a significant effect on *WL* and *SG*, water activity reduction and color/texture change. Regarding synergistic effects, interactions of temperature with glycerol concentration (for color) were found to have a significant effect on the parameters measured. The texture was positively affected by the OD temperature; an increase in OD temperature led to an increase in tomato firmness. The same approach for the modelling and validation of osmotic treatments of fruits (such as tomatoes) was used in the literature [6,7,9,23,24]. The regression equations (in uncoded, actual units) were as follows (Equations (13)–(17)):

Water activity a_w_ = 1.871 − 0.014408T − 0.001750t − 0.02115C + 0.000232T^2^ + 0.000017t^2^ + 0.000185C^2^ − 0.000007T ∗ t − 0.000032T ∗ C − 0.000003t ∗ C (13)

Water Loss *WL* = −95.8 + 0.643T + 0.044t + 2.887C − 0.00325T2 − 0.000560t2 − 0.02251C2 + 0.00133T ∗ t − 0.00602T ∗ C − 0.00022t ∗ C (14)

Solid Gain *SG* = 6.40 − 0.262T + 0.0183t − 0.085C + 0.00394T2 − 0.000061t2 + 0.00086C2 + 0.000146T ∗ t − 0.00008T ∗ C − 0.000255T ∗ C (15)

Color Δ*E* = −15.5 − 0.278T − 0.5008t + 1.386C − 0.00941T2 + 0.003228t2 − 0.01624C2 + 0.00109T ∗ t + 0.01511T ∗ C + 0.00110t ∗ C (16)

Texture *Firmness* = 12.90 − 0.210T − 0.3016t + 0.016C + 0.00374T2 + 0.001656t2 − 0.00055C2 + 0.000572T ∗ t − 0.00076T ∗ C + 0.001300t ∗ C (17)

**Figure 5:**
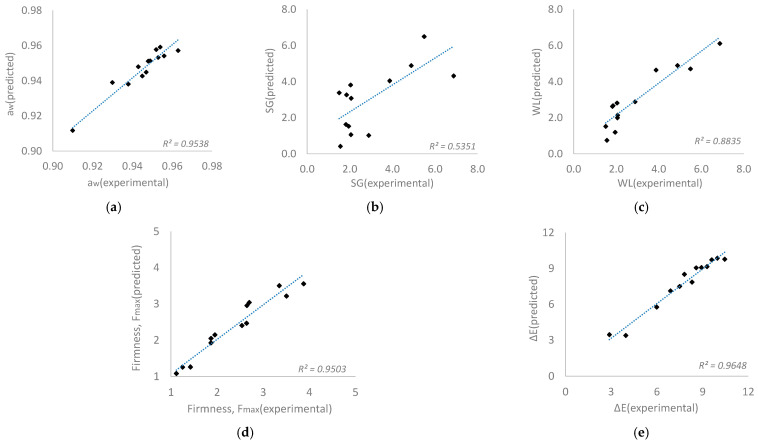
Linear correlation plots between predicted and observed values for (**a**) water activity (a_w_), (**b**) water loss (*WL*), (**c**) solid gain (*SG*), (**d**) color (Δ*E*) and (**e**) firmness (*F_max_*) responses.

In Figure 5, plots of the predicted and the observed (experimental) values are presented. An acceptable level of agreement (R^2^ > 0.8) was calculated. Moreover, based on the statistical results (ANOVA for Response Surface Quadratic Model, at a confidence level of 95%), it can be observed that *p*-value of the model, for all different indices investigated, is significant (*p* < 0.05), whereas *p*-value of lack of fit is non-significant (*p* > 0.05). From all the above information, it can be concluded that the developed models could adequately represent the data.

#### Osmotic Dehydration Process Optimization and Validation

**Figure 6:**
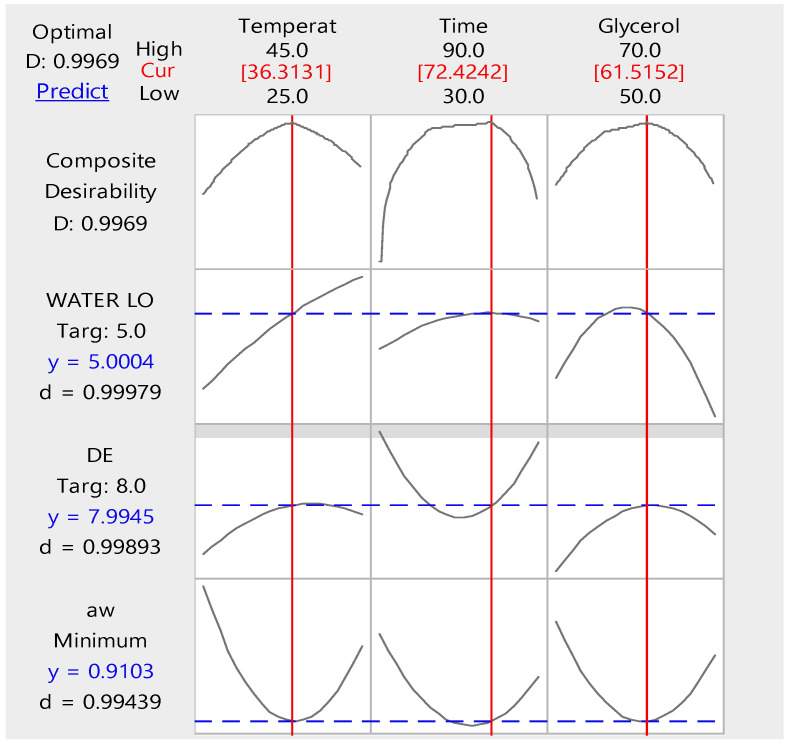
Desirability plot of variables.

According to mass transfer and quality loss results, of the osmodehydrated tomato (from the previous section), the criteria for the OD process optimization were set as: water loss *WL* ≤ 5, color change Δ*E* ≤ 8 and at the same time minimization of the water activity. The upper limit of acceptance for the color change was chosen with the help of the sensory evaluation test results as well as the kinetic study results for the color loss. The upper limit for the water loss was set based on the mass transfer kinetic modelling. To find the OD process parameters that meet the pre-determined criteria, the desirability function method was applied and the corresponding composite desirability profiles were depicted in Figure 5. To apply the methodology, for each of the critical process conditions (% glycerol, process temperature, process time,) the levels were allowed to take values within the experimental range. Optimal OD process conditions were found as follows: 36 °C–72 min, 61.5% glycerol, respectively, based on the simultaneous, multiple criteria set (Figure 6).

Figure 6 shows the desirability diagram of the process, with the criteria set for optimization. How effectively a combined variable satisfies the objectives is shown by the individual level of desirability (represented by d for each response) and the composite level of desirability (represented by D for the integrated response). While the composite factor (D) evaluates how effectively the arrangements optimize an integrated criterion, the individual desirability (d) evaluates the degree to which the arrangements optimize a particular response (y). The geometric mean of the individual desirabilities for the chosen responses is used to determine the composite desirability, in accordance with the algorithm used in this work. The composite desirability D = 0.99 of the configurations suggests that they significantly satisfy the required combination of criteria. Given that the individual desirability levels are d = 0.99, this is reasonable. In our study, the synthetic desirability (D = 0.99) indicates that the arrangements appear to significantly satisfy the combination of criteria required. This is reasonable since the individual desirability values are (d = 0.99).

In Table 3, predicted values of the dependent variables are estimated at those optimum conditions, calculated by the polynomial equations developed, and compared to values measured by an independent validation experiment (% Absolute Error < 20%). The data were validated by conducting an independent experimental test in the optimal process conditions (validation test). It is observed the % Absolute Error calculated for all measured values (*WL*, *SG*, a_w_, Δ*E*) (apart from the % Absolute Error for the texture) was below <20%. Various studies have shown that the variety of the tomato can affect its texture (firmness) after osmotic dehydration as different varieties have different textures, structures and chemical compositions. Some tomato varieties (depending on the growing conditions) may have thinner skin and softer cells, while others may have a denser structure or more flesh. These differences in structure and chemical composition can affect how the tomato responds to osmotic dehydration. Different quality parameters have also been reported for the same tomato varieties [52].

### 3.3. Shelf Life of Frozen Tomatoes, OD vs. Untreated, during Storage

#### 3.3.1. Quality Evolution during Storage at Isothermal Conditions

##### Physicochemical Characteristics

The osmodehydrofrozen cherry tomatoes had water activity values around 0.90 compared to 0.95 for the non-osmodehydrated frozen (untreated). The pH values were reduced from 4.10 to 3.90. After the application of osmotic dehydration, the soluble solids (expressed as °Brix) significantly increased, from 8.81 to 20.01. The frozen storage did not affect a_w_, pH, dry matter and soluble solids (*p* > 0.05).

##### Drip Loss and Texture

**Figure 7:**
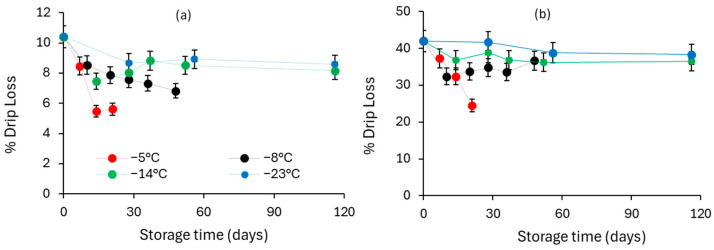
Evolution of drip loss (%) during storage for (**a**) non-pretreated and (**b**) OD-pretreated cherry tomatoes stored at T = −5, −8, −14, −23 °C (error bars indicate the standard deviation from replicates).

In Figure 7a,b the drip loss for untreated and OD-treated frozen cherry tomatoes stored at isothermal conditions is presented. Non-treated samples suffered from significantly higher drip loss and tissue softening at zero storage time. Tomato integrity was retained with storage time for all osmotically pre-treated samples (low drip loss showing a decrease with storage time and increased firmness not showing any clear tendency). Similar results were obtained for osmodehydrofrozen fruit and vegetables such as pineapple, mangoes melon and strawberries, respectively [39,53,54,55].

**Figure 8:**
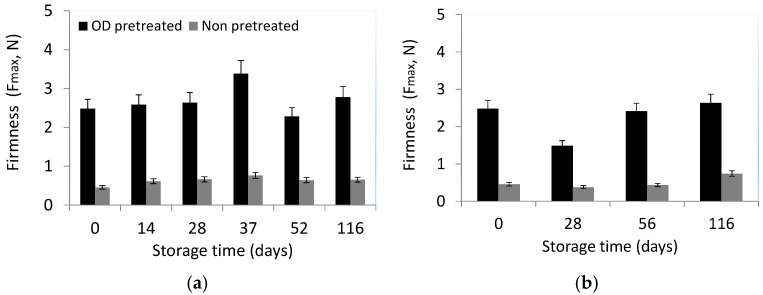
Evolution of firmness (*F_max_*, N) during storage for non-pretreated and OD-treated cherry tomatoes stored at (**a**) T = −14 °C and (**b**) T = −23 °C (error bars indicate the standard deviation from replicates).

The texture properties (mainly firmness) of tomato acceptability are significant. Changes in the composition and arrangement of the cells and the turgor during the maturation process lead to the development of firmness that impacts texture, shelf life, and total acceptance [56]. The firmness values for the osmodehydrated tomatoes were 2.664 ± 0.527 N at all storage temperatures compared to the firmness values of untreated tomatoes which were 0.527 ± 0.106 N. An increasing trend was observed during storage (not statistically significant). In Figure 8, the firmness values of untreated and OD-treated frozen cherry tomatoes stored at isothermal conditions T = −14 °C and −23 °C are representatively presented. The osmodehydrated tomatoes were characterized by the sensory panelists as “not soft”, and the same time as “having a more intense flavor, and a sweeter taste (acceptable and desirable)”. Osmodehydrated cherry tomatoes presented significantly increased firmness compared to their untreated counterparts (*p* < 0.05). Frozen delicate plant matrices, with high water content (such as tomatoes and fruits) pre-treated by osmotic dehydration have been reported to show improved texture, after thawing [39,55].

##### Color

The color of OD pretreated tomato samples did not present statistically significant differences compared to the color of untreated tomato samples. Color indices L, a, b of non-pretreated and OD pretreated tomato samples were measured as: Non: 28.49 ± 2.97, 18.18 ± 3.10, 13.65 ± 3.20; OD: 28.25 ± 3.41, 19.47 ± 1.30, 13.16 ± 1.82 (at storage time 0 day). According to Gould et al. [30], the color parameter a/b that exceeds the value 0.5 is accepted as a measure of ripeness. United States Department of Agriculture (USDA) recommendation [57], cherry tomatoes are considered attractive for marketing when their a/b ranges from 0.95 to 1.21. In this study, a/b values of untreated and OD pre-treated cherry tomatoes were calculated as 1.33 and 1.47, respectively.

**Figure 9:**
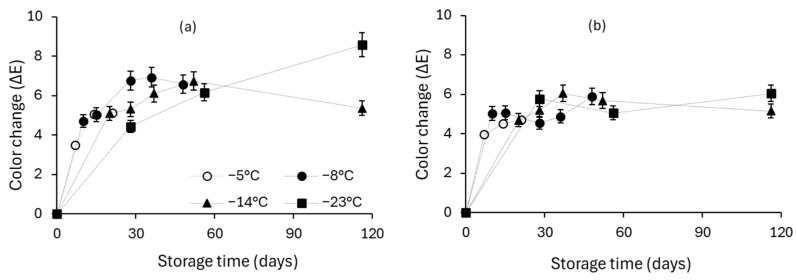
Evolution of color change (ΔΕ) during storage for (**a**) untreated and (**b**) osmodehyddrofrozen cherry tomatoes stored at T = −5, −8, −14, −23 °C (error bars indicate the standard deviation from replicates).

Cherry tomato color change during storage is attributed to lycopene degradation and pigment accumulation including anthocyanins, flavonoids and carotenoids in the tomato skin [47]. In this study, the total color change of all frozen cherry tomatoes was expressed by ∆*E* (Figure 9a,b). The storage of frozen cherry tomatoes significantly changed color. The color change was more intense for the osmotically dehydrated tomato samples. L and b parameters significantly increased; a parameter decreased. The same trend was observed for the non-pretreated tomato samples. Most published works reported a slight color change from red to orange. Urbanyi et al. (1998) reported a similar color change accompanied by a gradual reduction in carotenoids during 24 weeks of frozen storage [58]. The same was observed by Lisiewska et al. (2000) after 6 months of storage at −20 °C [29].

#### 3.3.2. Vitamin C, Lycopene and Sensory Quality Loss: Mathematical Modelling

**Figure 10:**
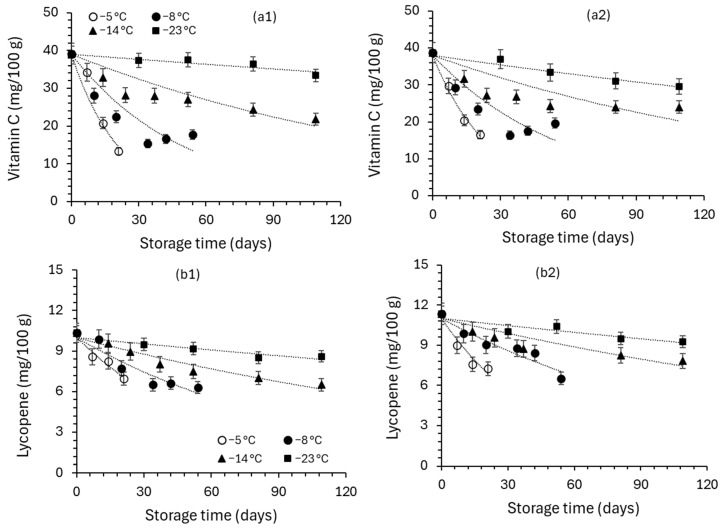
Evolution of (**a**) vitamin C and (**b**) lycopene during storage for (**1**) non-pretreated and (**2**) OD-pretreated cherry tomatoes stored at constant temperature conditions T = −5, −8, −14, −23 °C (error bars indicate the standard deviation from replicates).

The protective effect of OD on vitamin C, lycopene and sensory properties’ retention is demonstrated. Vitamin C, total lycopene and sensory quality of the non-pretreated and OD pre-treated cherry tomatoes stored at constant temperature conditions T = −5, −8, −14, −23 °C are presented (Figure 10). Vitamin C and lycopene, owing important health benefits, are considered quality indicators of cherry tomatoes [59]. As shown in Figure 10, throughout frozen storage, Vitamin C and lycopene content decreases. The decrease in Vitamin C and lycopene content can be attributed to oxidation [28]. Compared to the untreated samples, OD pre-treatment delayed the vitamin C and lycopene loss of cherry tomatoes during storage.

Osmodehydrated cherry tomatoes with glycerol-based solutions showed higher lycopene content than fresh cherry tomato, as reported by Heredia et al. [60,61]. According to Heredia et al. (2010), this could be due to the optimal temperature of synthesis of lycopene (40 °C), or to the effect of osmotic stress that encouraged phytochemicals generation and the presence of a precursor like sucrose [60]. Additionally, Heradia et al. [61] showed that the osmotic dehydration limited the isomerization during the later stage of drying, whereas both the total lycopene loss and the trans–cis isomerization, mainly to the 13-cis form, were favored by an increase in osmotic dehydration/drying temperature and the microwave power. In this study, the lycopene content of the osmodehydrated sample was 11.35 mg lycopene/100 g OD tomato compared to the value of the non-osmodehydrated tomato sample 10.35 mg/100 g. During frozen storage, after 3 months of storage at the temperature of −23 °C, the osmodehydrofrozen samples retained better their (total) lycopene content (83.1% of the initial content) compared to the non-osmodehydrofrozen tomato samples (81.5%, respectively of the initial content).

Dermesonlouoglou et al. [4] did not report statistically significant differences between untreated and OD pre-treated tomatoes, values ranged from 24.0 to 30.1 mg L-ascorbic acid/100 g OD tomato compared to the value of the untreated tomato 28.9 mg/100 g. After 12 months of storage at −20 °C, osmodehydrofrozen tomatoes retained better their vitamin content (66.2–88.0% of the initial) compared to the conventionally frozen, untreated and blanched tomato samples (44.1% and 57.8%, respectively). Osmodehydrofrozen tomatoes stored at higher temperatures (e.g., at −12 °C) also exhibited better Vitamin C stability for long storage times. In this study, the vitamin C content of osmodehydrated sample was 39.15 mg L-ascorbic acid/100 g OD tomato compared to the value of the non-osmodehydrated tomato sample 38.79 mg/100 g. During frozen storage, after 3 months of storage at the temperature of −23 °C, the osmodehydrofrozen samples retained better their vitamin content (85.0% of the initial vitamin content) compared to the non-osmodehydrofrozen tomato samples (76.2%, respectively, of the initial vitamin content). An et al., 2013 reported an improved retention of vitamin C in osmotically dehydrated and dried cherry tomatoes compared to dried untreated tomatoes [62]. However, a slight decrease in vitamin C after the osmodehydrofreezing process, possibly owing to a mild leakage of water-soluble compounds out of the cell tissue, has been reported [63]. Zhao et al. (2017) in [53] showed that when using a mixture of sucrose/glucose/fructose as the OD medium, the vitamin C of ODF mangoes was significantly decreased before frozen storage (with OD samples having a water content of approximately 80% w.b.). Nonetheless, as it was pointed out, this loss was counterbalanced by the improved vitamin C retention of OD samples during subsequent storage, possibly owing to rapid leaching via increased drip loss of the untreated samples [58]. Fan et al. (2020) studied osmotic dehydration with ultrasound enhancement before freezing of kiwi and found better retention of ascorbic acid content; however, they underlined that, since Vitamin C is a water-soluble compound, it can be readily lost during thawing, and thus the extent of drip loss in osmodehydrofrozen samples is strongly related to the retention of ascorbic acid [64]. Similar findings can be found in Xin et al. 2013, who observed a milder leak of vitamin C into the osmotic solution (containing 40% trehalose) when an ultrasound pretreatment was applied, mainly attributed to the shorter dehydration time compared with the OD counterparts (with a water content of approximately 65% w.b. [65].

In this study, OD frozen cherry tomatoes presented bright color, good texture and pleasant taste, whereas the untreated tomatoes suffered from a detrimental texture change [high drip loss (Figure 6) and tissue softening] and taste deterioration during storage. The pre-treated samples were preferred in terms of all sensory attributes including taste and they showed increased stability with storage compared to non-treated samples Dermesonlouoglou et al. (2008, 2016, 2018) reported that panel assessments confirmed the analytical results of better-quality retention of somodehydrofrozen strawberries, and kiwi, also judging their flavor as pleasant [55,66]. Other research results showed that osmotic dehydration enhanced the sensory attributes of frozen samples [64,67]. Zhao et al. (2016) found improved color and texture, reduced drip loss, as well as retention of the aromatic compounds in osmodehydrofrozen mangoes [68].

The kinetic models developed for frozen tomatoes, based on Equations (4) and (5), are presented in Table 4 (Equations (2)–(4)).

The rejection of both untreated and osmodehydrofrozen cherry tomatoes was due to sensory quality loss (the shelf life determining factor was the sensory evaluated overall impression). For example, the shelf life at −18 °C for untreated osmodehydrofrozen cherry tomatoes was calculated as: Untreated 42 days (sensory rejection), 166 days (vitamin C loss) and 212 days (lycopene loss) and OD pretreated: 136 days (sensory rejection), 208 days (lycopene loss) and 234 days (vitamin C loss), respectively. OD increased the shelf life of frozen cherry tomato (up to 3.5 times).

#### 3.3.3. Validation in Non-Isothermal Storage Conditions

Since storage and transportation temperatures of frozen foods (fruits) can vary, it is important to be able to reliably estimate the effect of this variability and predict product shelf life in the actual frozen food chain. The developed models at isothermal storage experiments were validated at non-isothermal (dynamic) storage conditions (fluctuating temperatures from −5 to −12 °C with effective temperature, T_eff_ = −7.3 °C) [37]. The results showed that these models can be adequately used at variable temperature conditions, within the same range and time of temperature fluctuations, to predict the tomato quality and consequently the remaining shelf life, based on the relative error (*RE*) values, calculated using Equation (5) (Table 5).

## 4. Conclusions

Osmotic dehydration could lead to adequate water removal, solids uptake and water activity reduction, while improving the retention of the main quality characteristics of frozen cherry tomato. Mass exchange (*WL*, *SG*, a_w_), firmness, and color change during OD were modeled using a second-order polynomial model describing the effect of the most important OD processing factors. The optimum processing conditions were OD: T_OD_ = 36 °C, t_OD_ = 72 min, and C_glyc_ + 61.5% *w*/*w*, based on the triple criterion of obtaining water loss *WL* ≤ 5, color change Δ*E* ≤ 8 and at the same time minimizing water activity at the end of the pretreatment. The shelf life determining criteria for both untreated and OD pretreated samples were found to be sensory rejection (expressed by the score given for the overall quality and acceptability). OD pretreated frozen cherry tomatoes presented an a_w_ decrease from 0.95 to 0.92, acceptable color, increased firmness, low drip loss and high vitamin C/lycopene retention during frozen storage. Based on this study, it is suggested that OD may have the potential as a pre-processing step in the manufacture of sensitive frozen fruit products. However, one should mention the limitations of the RSM implemented in the first part (OD process); being a local analysis, the developed response surface models, as outlined in Equations (13)–(17), are invalid for regions other than the studied ranges of independent factors investigated (temperature, glycerol concentration and osmosis time). Another often reported limitation is that Response Surface Methodology (RSM) is a ‘black box’ approach [69], and thus, estimating the accuracy of the approximation is rather difficult. Nonetheless, in our approach, an experimental validation of RSM models was performed at the optimal conditions calculated (to alleviate the acknowledged RSM weaknesses) and the applicability of the RSM equations derived was confirmed. Concerning the second part of the integrated analysis (the stability test during frozen storage), one should also bear in mind that the results of the Arrhenius equation should be cautiously used in different conditions, and always within the range of temperature conditions analyzed. Finally, one should also consider possible deviations from the Arrhenius law, if the glass transition of the frozen matrix occurs within the temperature range studied [70]. Especially in the particular study conducted, the tomato matrix was modified due to the OD process (solid impregnation and water activity decrease occurred; therefore, the glass transition temperature is expected to decrease, and thus a thorough investigation of kinetics at the lower temperatures of storage (<−18 °C) would be necessary to challenge the uniform application of the Arrhenius equation.

## Figures and Tables

**Table 1:** Actual and coded values of the investigated factors.

Independent OD Variable	OD Temperature (°C)	OD Time (min)	Glycerol Concentration (% *w*/*w*)	X_1_	X_2_	X_3_
High	25	30	50	+1	+1	+1
Center	35	60	60	0	0	0
Low	45	90	70	−1	−1	−1
Standard order	X_1_	X_2_	X_3_			
1	1	0	1			
2	−1	1	0			
3	0	−1	1			
4	0	1	−1			
5	1	−1	0			
6	1	0	−1			
7	−1	0	1			
8	−1	0	−1			
9	−1	−1	0			
10	0	0	0			
11	0	0	0			
12	0	1	1			
13	1	1	0			
14	0	−1	−1			
15	0	0	0			

**Table 2:** Water loss (*WL*), Solid Gain (*SG*), water activity (a_w_), hardness (*F_max_*) and color (Δ*Ε*) factor effects obtained from the second-order polynomial model for the osmo-dehydrated tomatoes (in coded coefficients).

Model Coefficient	*WL* (g w./g i.d.m.)	*SG* (g s./g i.d.m.)	a_w_	Firmness (*F_max_*, N)	Color (Δ*E*)
Constant a0	4.889 *	0.130	0.91000 *	1.253 *	7.498 *
Linear					
a1	1.340 *	0.1727	−0.00525	0.398 *	0.355
a2	0.317	0.0228	−0.00437	−0.147	−0.273
a3	−0.378	−0.0033	−0.00288	0.017	0.327
Quadratic					
a11	−0.325	0.394 *	0.02325 *	0.374	−0.9418 *
a22	−0.504	0.055	0.01550 *	1.490 *	2.906 *
a33	−2.251 *	0.086	0.01850 *	−0.055	−1.624 *
Interaction					
a12	0.400	0.044	−0.00225	0.172	0.327
a13	−0.602	−0.008	−0.00325	−0.076	1.511 *
a23	−0.067	−0.077	−0.00100	0.390	0.331
R^2^	0.9324	0.9211	0.9544	0.9503	0.9648
R^2^ (adj)	0.8835	0.8293	0.8722	0.8609	0.9013
*p*-value (model)	<0.05	<0.05	<0.05	<0.05	<0.05
Lack of fit (*p*-value)	>0.05	>0.05	>0.05	>0.05	>0.05

* Statistical analysis of the factors of polynomial equations (RSM) with variance analysis (ANOVA). *p* value < 0.05, values assigned an asterisk are statistically significant coefficients at a level of 0.95.

**Table 3:** Experimental and predicted values for the responses at optimum OD process parameters (temperature: 36 °C; time: 72 min; Glycerol concentration: 61.5% *w*/*w*) of cherry tomatoes.

	Experimental Value	Predicted Value	% Error
Water Loss (*WL*, g w./g i.d.m.)	4.881	4.890	0.21
Solid Gain (*SG*, g S./g i.d.m.)	0.130	0.152	16.5
Water activity (a_w_)	0.9105	0.9117	0.13
Color change (Δ*E*)	6.24	7.49	−18.0
Firmness (*F_max_*)	2.08	1.26	39.4

**Table 4:** Estimated kinetic parameters [*k_asc/lyc/sens_, k_ref_* (days^−1^) at *T_ref_* = −18 °C, *E_a_*, kJ/mol] for Vitamin C, lycopene and sensory quality of untreated and osmodehydrofrozen cherry tomatoes.

	Kinetic Model Structure and Model Dependent Variables	
	Untreated	OD Treated
Vitamin C *C_as_*_c_ = L-ascorbic acid content (mg/100 g)	*E_a_* = 88.8 kJ/mol *k_ref_* (−18 °C) = 0.0033 d^−1^ *SL* (50% loss): 20 d (−5 °C); 37 d (−8 °C); 87 d (−14 °C); 402 d (−23 °C)	*E_a_* = 115.6 kJ/mol *k_ref_* (−18 °C) = 0.0025 d^−1^ *SL* (50% loss): 15 d (−5 °C); 33 d (−8 °C); 102 d (−14 °C); 737 d (−23 °C)
Lycopene *C_lyc_* = Lycopene content (mg/100 g)	*E_a_* = 88.5 kJ/mol *k_ref_* (−18 °C) = 0.0025 d^−1^ *SL* (50% Loss): 44 d (−5 °C); 68 d (−8 °C); 130 d (−14 °C); 415 d (−23 °C)	*E_a_* = 86.1 kJ/mol *k_ref_* (−18 °C) = 0.0020 d^−1^ *SL* (50% Loss): 46 d (−5 °C); 70 d (−8 °C); 131 d (−14 °C); 395 d (−23 °C)
Sensory evaluation *S* = Score for sensory quality	*E_a_* = 56.58 kJ/mol *k_ref_* = 0.017 d^−1^ *S_i_* = 7.1, *S_f_* = 4 *SL*: 14 d (−5 °C); 19 d (−8 °C); 30 d (−14 °C); 67 d (−23 °C)	*E_a_* = 57.10 kJ/mol *k_ref_* = 0.009 d^−1^ *S_i_* = 9.0; *S_f_* = 4 *SL*: 50 d (−5 °C); 66 d (−8 °C); 100 d (−14 °C); 207 d (−23 °C)

**Table 5:** Quality loss rates (*k*), experimental and predicted, for Vitamin C, lycopene and sensory quality of untreated and OD-treated cherry tomato samples, and the relative error (RE) values.

	Vitamin C		Lycopene		Sensory Quality	
	Untreated	OD	Untreated	OD	Untreated	OD
k_experimental_ (d^−1^)	0.0179	0.0228	0.011	0.011	0.1816	0.0760
k_predicted_ (d^−1^)	0.023 ± 0.023	0.0284 ± 0.019	0.0074 ± 0.0052	0.0123 ± 0.0143	0.1867 ± 0.247	0.0654 ± 0.0305
%RE	20.01	19.71	−47.05	10.56	2.73	−16.2

## Data Availability

The original contributions presented in the study are included in the article, further inquiries can be directed to the corresponding author.
